# Pre-pandemic artificial MERS analog of polyfunctional SARS-CoV-2 S1/S2 furin cleavage site domain is unique among spike proteins of genus *Betacoronavirus*

**DOI:** 10.1186/s12863-024-01290-2

**Published:** 2024-12-17

**Authors:** Andreas Martin Lisewski

**Affiliations:** School of Science, Constructor University, 28759 Bremen, Germany

**Keywords:** Genomics, Directed evolution, Artificial virus host, Betacoronavirus, Furin cleavage site, Nuclear localization signal, O-glycosylation

## Abstract

**Objectives:**

SARS-CoV-2 spike (S) glycoprotein furin cleavage site is a key determinant of SARS-CoV-2 virulence and COVID-19 pathogencity. Located at the S1/S2 junction, it is unique among sarbecoviruses but frequently found among betacoronaviruses. Recent evidence suggests that this site includes two additional functional motifs: a pat7 nuclear localization signal and two flanking *O*-glycosites. However, a systematic genus and subgenus analysis of spike protein sequences bearing this polyfunctional sequence domain has been missing.

**Data description:**

Here we report comprehensive sequence data to demonstrate that among spike proteins of genus *Betacoronavirus* and outside of the SARS-CoV-2 clade a fully analogous S1/S2 domain was found in only one other virus: the artificial MERS infectious clone MERS-MA30, described already in 2017, which was rationally selected from serial passage in genetically humanized mice. As the evolutionarily closest betacoronaviruses outside of the SARS-CoV-2 clade lack all its three functional motifs, these data extend—beyond natural evolution and zoonosis—the current view on SARS-CoV-2 pre-pandemic origins by presenting the analogous S1/S2 MERS-MA30 sequence domain as a precise molecular blueprint for SARS-CoV-2.

## Objective

The furin cleavage site (FCS) at the S1/S2 domain junction of the SARS-CoV-2 spike (S) glycoprotein has been recurrently discussed in the context of SARS-CoV-2 origins, SARS-CoV-2 virulence, and COVID-19 pathogenicity [1, 2]. In comparison to bat coronavirus RaTG13 (GenBank: https://identifiers.org/nucleotide: MN996532) and BANAL-20-52 (GenBank: https://identifiers.org/nucleotide: MZ937000), the closest genomic betacoronavirus relatives to SARS-CoV-2, the reference sequence (Wuhan-Hu-1 isolate, GenBank: https://identifiers.org/nucleotide: NC_045512) features a four amino acid ^681^PRRA^684^ insert between two adjacent Ser and Arg residues, resulting in a RXXR minimal FCS. This FCS, which does not fully match the canonical FCS motif RX(K/R)R (see [1]), has not been seen in other sarbecoviruses [3]; on the other hand, simple furin-like cleavage sites at S1/S2 domains in other betacoronavirus spike glycoproteins have been identified and used as evidence for an entirely natural origin of SARS-CoV-2 [1, 4, 5].

The novel S1/S2 FCS [6] is flanked by two proximal *O*-linked glycosylation sites, Thr678 and Ser686 [7, 8]. *O*-linked glycosylation of these two residues demonstrated their functional role as modulators of FCS, membrane fusion, and virus penetration activity [7, 9–11]. In parallel, Hatmal and colleagues [12] predicted already in 2020 that this FCS itself is part of a pat7 nuclear localization signal (NLS) at the S1/S2 domain junction of SARS-CoV-2 spike, ^681^PRRARSV^687^. This was consistent with later observations of the spike glycoprotein localizing at and inside the nucleus during SARS-CoV-2 infection and COVID-19 progression [13–15]. Sattar et al. more recently confirmed the ^681^PRRARSV^687^ S1/S2 NLS [16] and showed that SARS-CoV-2 spike translocated into the nucleus whereas a pat7 deficient SARS-CoV spike did not. As one of the classical NLS [17], pat7 is defined by seven consecutive residues starting with a Pro, followed by a stretch of four residues that include three basic amino acids [18]. The spike SARS-CoV-2 S1/S2 junction domain between residues Thr678 and Val687 is therefore polyfunctional bearing at least the above three functional sequence motifs. The following data represents a comprehensive search for such S1/S2 polyfunctional domains among other spike protein sequences of genus *Betacoronavirus*.

## Data description

To detect sequence analogues of the S1/S2 polyfunctional domain across a comprehensive set of relevant virus species, we initially turned to the curated ‘*betacoronavirus spike glycoprotein*’ InterPro/UniProt collection of overlapping homologous superfamilies (Table 1, Data file 1 [19], InterPro: https://identifiers.org/interpro: IPR042578). From it all predicted FCS motifs within a constant frame of twenty amino acid residues [20] were extracted (Data file 2 [19]), which in a second step were automatically filtered for pat7 NLS motifs. After removing sequence fragments and duplicates, this procedure resulted in a set of twenty representative sequences (Data file 3 [19], and Data file 4 [19]; numbers 1–19 and 21 in tables) with two betacoronavirus positive hits outside of the SARS-CoV-2 clade: one synthetic merbecovirus MERS-MA30 (Data file 3, number 19 [19]; GenBank: https://identifiers.org/nucleotide: MT576585), which was passaged, rationally selected and cloned from an artificial host (transgenic human dipeptidyl peptidase 4 receptor knockin mice that permit viral entry) [21]; and one human embecovirus HCoV-HKU1 (Data file 3, number 21 [19]; GenBank: https://identifiers.org/nucleotide: DQ415902.1). Two closely related betacoronavirus sequences were manually added as pat7 negatives (Data file 3, numbers 20 and 22; [19]): the spike protein sequence from the closest natural parental strain of MERS-MA30, i.e. the original MERS CoV (human betacoronavirus 2c isolate EMC/2012; GenBank: https://identifiers.org/ncbiprotein:AFS88936.1); and the aligned spike protein sequence domain from the non-human betacoronavirus genomically closest to SARS-CoV-2, i.e. the bat coronavirus RaTG13 (GenBank: https://identifiers.org/ncbiprotein: QHR63300.2).

Within this set of spike S1/S2 sequences, pat7 nuclear localization signals were detected (Data file 3 [19]; and Data file 5 [19]) in SARS-CoV-2 spike S1/S2 (including the original reference sequence from Wuhan-Hu-1), in the S1/S2 sequence of MERS-MA30 CoV (GenBank: https://identifiers.org/ncbiprotein: QKX95939.1, and in the human coronavirus HKU1 S1/S2 spike (GenBank: https://identifiers.org/ncbiprotein: ABD75545.1). In MERS-MA30 S1/S2, pat7 NLS was not the product of natural evolution, but of the MERS (isolate EMC/2012) parental S1/S2 sequence ^744^TLTPRSVRSV^753^ change through an adaptive mutation Ser749Arg on an artificial genetic background rationally selected, after serial passage in transgenic murine hosts, for genomic stability [21].

As further verification of the proposed analogy between SARS-CoV-2 and MERS-MA30 CoV, prior experimental evidence for the flanking SARS-CoV-2 Thr/Ser *O*-glycosite residue pair was homology inferred within the SARS-CoV-2 clade, and tested with the standard prediction software NetOGlyc4.0 [22]. In a resulting positive validation, these flanking *O*-glycosite residues were confirmed for SARS-CoV-2 spike Thr678 and for Ser686; and robustly predicted for MERS-MA30 and MERS at the corresponding spike residues Thr744 and Ser752 (see, Data file 3 and Data file 6 [19]).

To test the sensitivity of these sequence hits on the size of the sequence search space, the output in Data File 3 was also independently verified through NCBI blastp searches, across all 10,766 betacoronavirus protein sequences outside of the SARS-CoV-2 clade in that database. This number was an order of magnitude larger than the 1,179 betacoronavirus sequences of that kind used above. The test result supported (Data file 7 [19]) the non-random and spike S1/S2 specific pat7/FCS motif design as no other spike sequence motif representatives were found than those already given (Data file 3 [19], numbers 19 and 21).

The other candidate polyfunctional betacoronavirus sequence detected outside of the SARS-CoV-2 clade was the S1/S2 spike domain from human coronavirus HKU1 (number 21 in Data file 3 [19]). Its sequence presented the canonical FCS motif RRKR embedded into a complete pat7 motif, ^749^PSSRRKR^755^; however, there was no Thr/Ser glycosite residue pair at or near the expected flanking positions, and therefore the sequence was not a complete functional analog of the corresponding SARS-CoV-2 domain. This negative result was confirmed with NetOGlyc4.0 (see, Data file 6 [19]). By contrast, the MERS-MA30 spike S1/S2 sequence comprised the entire polyfunctional domain, ^744^TLTPRRVRSV^753^, with pat7 and stable *O*-glycosite predictions for the consensus residues Thr744 and Ser752 (see, Data file 6 [19]). Also, unlike SARS-CoV-2 and MERS-MA30 CoV, in the HKU1 sequence any FCS dependent proteolytic cut would be outside of the pat7 NLS, due to a double amino acid downstream shift of the FCS sequence location, leaving pat7 entirely within S1. Functionally, this difference directly implies that after S1/S2 cleavage HKU1 S1, but not SARS-CoV-2/MERS-MA30 S1 or S2, retain this pat7 NLS. In a further genetic difference, while a loss of the multibasic RXXR FCS would abrogate pat7 in the SARS-CoV-2/MERS-MA30 consensus motif, PRRXRSX, in HKU1 CoV S such motif interlocking is not observed: for example, a change of the furin cleavage site’s first or last Arg into a non-basic residue would preserve pat7 in PSSRRKR.

In the same data set (Data file 3 [19]), SARS-CoV-2 spike protein sequences 1–18 corresponded to within-clade SARS-CoV-2 genomic variants that tightly preserved the entire polyfunctional TXXPRRXRSX S1/S2 consensus sequence. When ordered by their sequence similarity distance to the MERS-MA30 S1/S2 domain, two early SARS-CoV-2 pre-pandemic variants (i.e., variants collected in March 2020, or earlier) were the closest to MERS-MA30 S1/S2: the A684V SARS-CoV-2 variant (number 17 in Data file 3 [19], first isolated in Saudia Arabia, Jeddah, in March 2020), which identically shared the pat7 and the predicted *O*-glycosite pair; and the rarer A684V/A688P double variant (number 18 in Data file 3 [19], globally isolated only once in Iran during March of 2020), which by the A688P mutation was closer to the MERS-MA30 S1/S2 sequence than the A684V single variant. Of note, the parental MERS betacoronavirus first originated in Saudi Arabia, Jeddah region, in 2012; and the March 2020 SARS-CoV-2 outbreaks in Saudi Arabia and in Iran were linked epidemiologically: the Saudi Arabia index case from early March 2020 was a Saudi traveler who had returned from Iran [23]. These phylogenetic data indicate that, when MERS-MA30 S1/S2 was provisionally positioned as an ancestral genomic reference, the corresponding polyfunctional SARS-CoV-2 S1/S2 domain led to a specific prediction about the geographic (Saudi Arabia, Jeddah region; or Iran) and temporal (March 2020, or before) origin of a rare pre-pandemic SARS-CoV-2 genomic variant of epidemiologic interest (spike A684V or A684V/A688P).

Collectively, these data suggest that, within genus *Betacoronavirus*, MERS-MA30 S1/S2 spike—a year 2017 or earlier product of directed adaptation and rational selection in an artificial (i.e., genetically engineered) murine host—is the only instance of a complete pat7/FCS/*O*-glycosite composite motif fully analogous to the S1/S2 polyfunctional spike sequence domain of SARS-CoV-2.

**Table 1:** Overview of data files/data sets

Label	Name of data file/data set	File types (file extension)	Data repository and identifier (DOI or accession number)
Data file 1	betacov_protein_matching_IPR042578.fasta	fasta file (.fasta)	Zenodo (10.5281/zenodo.14476845) [19]
Data file 2	betacov_protein_matching_IPR042578_motif.fasta	fasta file (.fasta)	Zenodo (10.5281/zenodo.14476845) [19]
Data file 3	table_s1s2_hits_betacov_polyf.pdf	Table (.pdf)	Zenodo (10.5281/zenodo.14476845) [19]
Data file 4	table_s1s2_hits_betacov_polyf.xlsx	Excel table (.xlsx)	Zenodo (10.5281/zenodo.14476845) [19]
Data file 5	betacov_s_s1s2_pat7_nls_psort.txt	Text file (.txt)	Zenodo (10.5281/zenodo.14476845) [19]
Data file 6	betacov_s_s1s2_oglyc_netogly.txt	Text file (.txt)	Zenodo (10.5281/zenodo.14476845) [19]
Data file 7	betacov_s1s2_nls_pat7_furin_blastp.txt	Text file (.txt)	Zenodo (10.5281/zenodo.14476845) [19]

## Limitations

The pre-pandemic MERS-MA30 CoV S1/S2 junction domain is a precise sequence analog of the corresponding SARS-CoV-2 S1/S2 polyfunctional domain; however, to further confirm this analogy, it would be necessary to show in experiment that the viral pat7 detected in MERS-MA30 CoV S1/S2 is a functional NLS, and that the predicted flanking *O*-glycosites Thr744 and Ser752 are glycosylated during the infectious cycle.

In addition to this unique MERS-MA30 spike protein domain from an artificial betacoronavirus, continued sequencing of environmental coronavirus samples might still identify natural betacoronavirus spikes with a combined pat7/FCS and *O*-glycosylated S1/S2 structural motif fully analogous to SARS-CoV-2 S1/S2. Until such sequence of natural origin is reported, the current data contrasts preliminary analyses [1, 4, 5] which claimed that the simple SARS-CoV-2 S1/S2 FCS, along with similar simple FCS found in other betacoronaviruses, provide already sufficient evidence for its natural evolutionary origin.

## Data Availability

The data described in this Data Note can be freely and openly accessed on *Zenodo* (*zenodo.org*) repository under (10.5281/zenodo.14476845). See Table 1 for details and links to the data [19].

## Abbreviations

CoV: Coronavirus
SARS: Severe acute respiratory syndrome
MERS: Middle-east respiratory syndrome
HCoV-HKU1: Human betacoronavirus hongkongense
S: Spike protein
FCS: Furin cleavage site
NLS: Nuclear localization signal
pat7: Pattern 7
MA30: Murine adapted in 30 passages
